# Parent–Child Relationship Typologies and Associated Health Status Among Older Adults in the United States and China: A Cross-Cultural Comparison

**DOI:** 10.1093/geroni/igae050

**Published:** 2024-05-18

**Authors:** Dexia Kong, Peiyi Lu, Bei Wu, Merril Silverstein

**Affiliations:** Department of Social Work, The Chinese University of Hong Kong, Sha Tin, Hong Kong SAR, China; Department of Social Work and Social Administration, The University of Hong Kong, Pok Fu Lam, Hong Kong SAR, China; Rory Meyers College of Nursing, New York University, New York, New York, USA; Departments of Sociology, Maxwell School, Syracuse University, Syracuse, New York, USA; Department of Human Development and Family Science, Falk College, Syracuse University, Syracuse, New York, USA

**Keywords:** Cognition, Cultural differences, Depressive symptoms, Functional limitations, Intergenerational relationship

## Abstract

**Background and Objectives:**

Cultural differences in intergenerational relationships have been well established in prior research. However, cross-national comparison evidence on the parent–child relationship and its health implications remains limited.

**Research Design and Methods:**

Data from the 2014 U.S. Health and Retirement Study and the 2015 Health and Retirement Longitudinal Study in China were used (*N*_US, non-Hispanic Whites only_ = 3,918; *N*_China_ = 4,058). Relationship indicators included coresidence, living nearby, having weekly contact, receiving assistance with daily activities, providing grandchild care, and financial transfer to/from children. Latent class and regression analyses were conducted.

**Results:**

Four classes were identified for non-Hispanic White older Americans: (1) distant and uninvolved (6.58%), (2) geographically proximate with frequent contact and downward support (47.04%), (3) coresident with frequent contact and upward support (13.1%), and (4) geographically proximate with frequent contact (33.28%). Three classes were identified among older Chinese: (1) coresident with frequent contact and upward support (37.46%), (2) coresident/interdependent (25.65%), and (3) geographically proximate with frequent contact and upward financial support (36.89%). For non-Hispanic White older Americans, providing downward support was associated with fewer functional limitations and better cognition. Receiving instrumental support from children was associated with more depressive symptoms, more functional limitations, and poorer cognition among older Chinese.

**Discussion and Implications:**

Cultural contrasts were evident in parent–child relationship typologies and their health implications. Compared to the U.S. non-Hispanic Whites, parent–child relationships in China tended to be closer and associated with poorer health status. The findings call for culturally relevant strategies to improve parent–child relationships and ultimately promote the health of older adults.

**Translational Significance:** This is the first cross-cultural comparative study examining the latent typologies of multifaceted parent–child relationships and their associated health status in the United States and China. Cultural contrasts were evident in the typologies of parent–child relationships. Moreover, various parent–child relationship typologies have differing associations with health among older Chinese and American adults. The findings highlight the need for culturally relevant strategies to improve parent–child relationships and ultimately promote health among older adults in different sociocultural contexts.

Intergenerational parent–child relationships, which have been associated with the health and well-being of older adults (Antonucci et al., 2011; Lowenstein, 2007; Silverstein et al., 2010), are embedded in and shaped by larger societal and cultural contexts (Antonucci & Jackson, 2007; Silverstein & Giarrusso, 2010). For instance, individualistic cultures emphasize individual independence, whereas collectivist cultures value interpersonal connectedness and harmonious interdependence among individuals (especially family members; Markus & Kitayama, 1991). Although many studies have examined the intergenerational relationship in various Western countries like the United States (Guo et al., 2020), or Eastern countries like China (Guo et al., 2012), most have focused on a single country. A few cross-national studies have conducted comparisons within the same cultural setting, such as the United States and European countries dominated by individualism (Dykstra & Fokkema, 2011; Katz et al., 2015; Lowenstein, 2007; Silverstein et al., 2010), or East Asian countries influenced by collectivism (i.e., China, Korea, and Japan; Lin & Yi, 2013). Thus far, no prior studies have compared intergenerational relationships between the United States and China, representing individualistic and collectivist cultures, respectively.

Moreover, previous research has primarily focused on the significant determinants/predictors of different intergenerational relationship types (Guo et al., 2018), whereas the associated health implications have received less attention. Using nationwide population-based cohort datasets from the United States and China, this study aims to (a) advance the understanding of parent–child relationships by classifying their underlying structures in two different cultural contexts; and (b) investigate their association with multiple health domains (i.e., depressive symptoms, functional limitations, and cognition).

## Intergenerational Relationships in the United States and China

The intergenerational solidarity model (Bengtson & Roberts, 1991; Silverstein & Bengtson, 1997) is a guiding framework for comprehending the intricate dynamics and patterns of intergenerational relationships. According to the model, intergenerational relations encompass six key dimensions: association (frequency and types of intergenerational interactions/contact), affection (sentiment and reciprocity among intergenerational members), consensus (intergenerational agreement on values, attitudes, and beliefs), function (frequency and reciprocity of intergenerational sharing of resources), norms (endorsement of intergenerational roles and expectations), and structure (number, type, and geographic proximity of intergenerational members).

Informed by the solidarity model, intergenerational relationships have been examined in prior research. A review of the existing literature reveals that intergenerational relationships in the Western cultural context emphasize independence between generations (Logan & Bian, 2004; Swartz, 2009). Specifically, parent–child relationships in the United States are often characterized by the residential independence between older parents and their adult children. Coresidence and assistance occurred in some special situations, such as aging parents experiencing poor health conditions or adult children having financial difficulties (Lee & Kim, 2022; Silverstein et al., 2006; Swartz, 2009; Xu et al., 2019). In these cases, older parents and their adult children might choose to live together and support each other. Such functional exchanges (i.e., financial and instrumental support) typically occur on an as-needed basis rather than in the form of routine mutual support (Silverstein & Giarrusso, 2010; Swartz, 2009). Regardless of geographical distance, many older Americans have frequent contact and close emotional connections with their adult children (Silverstein & Giarrusso, 2010; Swartz, 2009). In individualistic societies like the United States, aging parents tend to provide adult children with more assistance than vice versa, and have limited expectations of upward assistance (Nauck & Suckow, 2006). Overall, intergenerational ties in the United States are driven by independence and self-sufficiency.

In contrast, China’s relational/collectivist culture emphasizes interdependence and mutual support among family members (Campos & Kim, 2017). In particular, filial piety, which prescribes caring for aging parents as a familial obligation and responsibility of adult children, represents the fundamental value governing parent–child relationships in China (Shi, 2009). The Law on Protection of the Rights and Interests of Older Persons mandates caring for aging parents as a legal responsibility of adult children (Chou, 2011), so coresidence and providing support for older parents are prevalent in China, especially in rural regions (Zhang et al., 2014). In addition, community-based care services remain unavailable or inaccessible to most people (Peng & Wu, 2021), so older parents are often cared for by their adult children (Lin & Yi, 2013; Zhu, 2016). Healthy older people may also choose to move in to help care for grandchildren, and physically frail older people may live with their children to receive instrumental care (Zeng & Xie, 2014; Zimmer & Korinek, 2010). Intergenerational exchanges primarily flow from adult children to aging parents, rather than vice versa (Logan & Bian, 2004; Nauck & Suckow, 2006).

Taken together, parent–child relationships in the United States are characterized by independence, whereas those in China emphasize interdependence with a cultural norm of mutual support (Logan & Bian, 2004; Nauck & Suckow, 2006). Given the cultural differences, an expanded understanding of intergenerational relationship typologies in the United States and China, which represent individualistic and collectivist societies, respectively, can extend the literature by contributing cross-cultural evidence.

## Intergenerational Relationships and Health in Later Life

Recent theories propose three pathways through which social relationships (including intergenerational relationships) can influence an individual’s health. First, intergenerational relations influence health via behavioral pathways. For instance, as individuals receive more intergenerational support, they are less likely to engage in health-damaging behaviors (e.g., excessive alcohol consumption and physical inactivity; Umberson & Karas Montez, 2010). Second, intergenerational support can influence health via psychological pathways. A higher level of positive intergenerational support is associated with positive psychological states, such as enhanced self-efficacy, self-worth, and sense of well-being, all of which are closely connected to health (Berkman et al., 2000). Third, intergenerational relationships can influence health via physiological pathways. Specifically, supportive intergenerational exchanges can decrease allostatic load and promote immunological functioning (Umberson & Karas Montez, 2010).

Campos and Kim’s (2017) theoretical model of cultural effects on the links between social relationships and health further identifies the crucial role of culture in shaping intergenerational relationships. Substantial cross-cultural heterogeneities in the function, quantity, frequency, and source of support are anticipated in intergenerational exchanges, which subsequently result in distinct health outcomes among older adults in various cultural settings (Campos & Kim, 2017).

Collectively, these theories suggest that the potential health impacts of intergenerational parent–child relationships are multifaceted and can differ across cultures. Previous studies concerning intergenerational relationship typologies have primarily focused on the significant determinants of the typologies (Guo et al., 2018), whereas the associated health statuses have received less attention. Some studies investigating the association between intergenerational relationship typologies and health have focused on a single health variable, such as psychological well-being (Kim et al., 2019), or cognitive function (Li et al., 2021), restricting cross-study comparisons. To expand our understanding of the link between relationship typologies and health, examining multiple health indicators simultaneously is warranted. In this study, we conceptualized health as a multidimensional concept encompassing physical, psychological, and cognitive well-being.

## Research Objectives

By leveraging nationwide population-based data from the United States and China, this study aimed to extend existing evidence in two critical ways. First, we used latent class analysis (LCA) to characterize latent typologies of parent–child relationships in two unique sociocultural contexts (i.e., the United States and China). LCA can depict the complexity of parent–child relationships in different sociocultural contexts, which addresses the need to use sophisticated approaches to capture the diversity and fluidity of contemporary family relationships (Silverstein et al., 2006). Second, after identifying the latent typologies in the two countries, we examined their associations with multiple aspects of health (i.e., depressive symptoms, functional limitations, and cognitive function) to achieve a comprehensive understanding of the health impacts. Ultimately, the study findings have the potential to inform strategic recommendations for developing culturally relevant interventions for family relationships and ultimately health promotion in later life.

## Method

### Data and Sample

Cross-sectional data from the 2014 U.S. Health and Retirement Study (HRS) and the 2015 China Health and Retirement Longitudinal Survey (CHARLS) were used. The HRS is one of the most widely used population-based cohort studies of American older adults. Since 1992, the HRS has surveyed about 20,000 American older adults aged 50 and above nationwide every 2 years. It covers a wide range of topics, including demographic characteristics and family, health, and financial status (HRS, 2022). A user-friendly longitudinal version of the HRS is provided by the RAND Corporation (Bugliari et al., 2018, 2021). CHARLS is an international HRS family study conducted in China. Since it was initiated in 2011, about 17,500 Chinese people aged 45 and over have been surveyed every 2 or 3 years (CHARLS, 2022). Because HRS and its international family studies were designed with cross-national comparability in mind, leveraging these data sets enables valid inferences regarding the differences between countries in this study. The Gateway to Global Aging Data team at the University of Southern California further harmonizes the coding of HRS and CHARLS to facilitate cross-national comparisons (g2aging, 2022).

This study used data from multiple sources, including the raw core HRS, RAND-HRS, and harmonized CHARLS data. We used the cross-sectional data in 2014–2015 because it was the latest harmonized data set in the two countries with all three health measures at the time of data analysis (February 2022). We restricted our sample to older adults aged 65 and above with at least one living child. Although China has 56 ethnic groups, the country’s culture is dominated by Confucianism and shared by all ethnic groups, so all Chinese respondents were included in the analysis regardless of ethnicity (*N* = 4,058). In contrast, the United States is a country with diverse races/ethnicities with different cultures, and parent–child relationships are influenced by individual cultural backgrounds. In particular, European Protestants have distinct familial patterns from those of East Asian and Latino descent (Campos & Kim, 2017). Because the HRS study had much smaller sample sizes in racial/ethnic minority groups, this study focuses on non-Hispanic White respondents (*N* = 3,918). As such, we contrasted Western culture (non-Hispanic White Americans as an example) with Eastern culture (Chinese as an example). As a sensitivity check, we repeated the analyses on all U.S. racial/ethnic samples (*N* = 5,546).

### Measures

A detailed list of measures used in this study is provided in Supplementary Table 1.

#### Parent–child relationship indicators

Seven parent–child relationship indicators were included in the analyses based on prior research (Guo et al., 2020; Lu et al., 2021; Silverstein & Bengtson, 1997) and their availability in both the HRS and CHARLS (Bugliari et al., 2018; Phillips et al., 2021). Respondents were asked if they (a) coresided with their children; (b) lived nearby their children (if the respondents did not coreside with their children, they were then asked if they lived near their children. Nearby was defined as within 10 miles in HRS and the same city/county in CHARLS); (c) had weekly contact with their children (either in-person or by phone or email/mail); (d) received instrumental assistance (i.e., help with activities of daily living or instrumental activities of daily living) from their children; (e) provided grandchild care (defined as ≥100 hrs in the past 2 years); (f) received any financial transfer from their children; and (g) provided any financial transfers to their children. All indicators were binary (0 = no, 1 = yes). Emotional affectual indicators (e.g., emotional support) were unavailable in CHARLS and were thus excluded from the analysis to maintain cross-national consistency.

#### Health status

Three health statuses were included in this study. Depressive symptoms were measured using the Center for Epidemiological Studies—Depression Scale (CES-D; Kohout et al., 1993; Radloff, 1977). The CES-D contains 8 items in HRS and 10 items in CHARLS. The total CES-D score is calculated by adding up all items. The CES-D has been shown to be a reliable and valid tool for screening depression among community-dwelling residents and was used in both the HRS and CHARLS (Lei et al., 2014; Wallace & Herzog, 1995). Functional limitation measured the extent of difficulty respondents experienced in performing certain physical tasks (e.g., walk several blocks, jog 1 mile, sit for 2 hrs; Guralnik & LaCroix, 1992). There are 12 items in the HRS and 9 items in the CHARLS. The functional limitation measures have also been validated and widely used in both Chinese and American populations (Wallace & Herzog, 1995; Wu et al., 2018).

Cognitive function was measured using the sum of memory and mental status measures (Dixon et al., 1988; O’Hara et al., 1986). Memory evaluated the respondents’ ability to recall a list of words immediately and after a delay (20 items in both HRS and CHARLS). Mental status assessed the respondents’ capacity to complete several tasks, including serial 7s, backward counting from 20, and naming dates and objects (15 items in HRS and 10 items in CHARLS). The total cognitive function score was calculated by adding the scores for memory and mental status. The cognition measures were evaluated as reliable and valid in two surveys (Lei et al., 2012; McArdle et al., 2007). To facilitate cross-country comparison and interpretation (Díaz-Venegas et al., 2018), we rescaled each health measure to range from 0 to 1, so that a higher score indicates more depressive symptoms, more functional limitations, or better cognitive function (Supplementary Table 1).

#### Covariates

Several individual-level demographic and health characteristics and household-level factors were included as covariates because they have been linked to both the independent variable (i.e., parent–child relationship) and dependent variable (i.e., older adults’ health) , as shown in prior research (Chen & Silverstein, 2000; Guo et al., 2018, 2020; Lu et al., 2021). Individual-level demographic variables included age (continuous), gender (female/male), education (based on the 1997 International Standard Classification of Education codes), marital status (partnered/partnerless), and retirement status (yes/no). Individual-level health characteristics were self-reported health (ordinal; from 1 = very poor to 5 = very good) and number of self-reported doctor-diagnosed chronic diseases (including high blood pressure, diabetes, cancer, lung disease, heart problem, stroke, psychiatric problems, arthritis; range 0–8). Household-level covariates were household size (number of people living in the household), number of children (count), number of grandchildren (count), and household income (transformed into purchasing power [unit: U.S. dollars] and then log-transformed).

### Data Analysis

**Figure 1.**
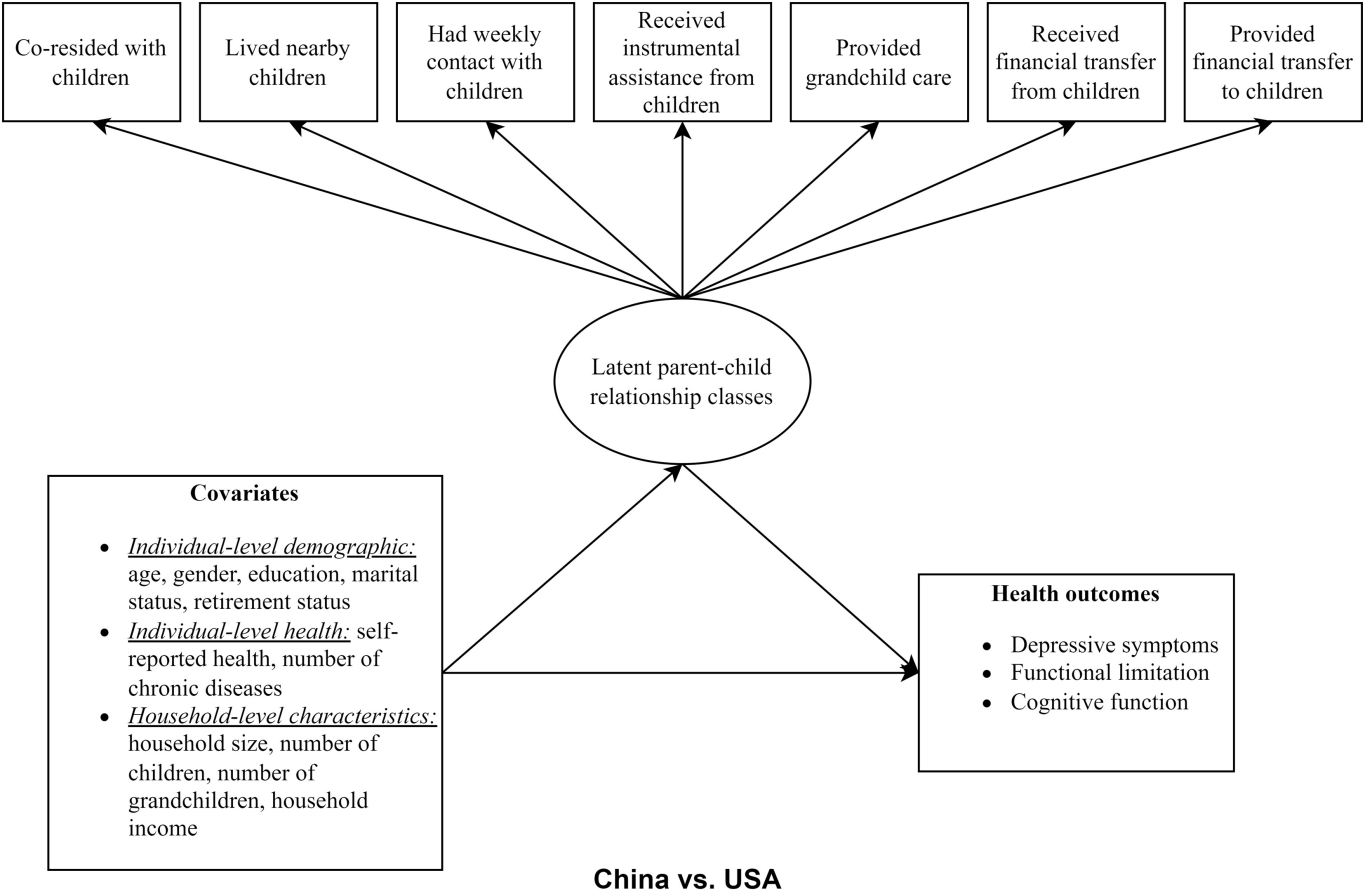
Statistical and conceptual framework for this study.

We first described and compared the characteristics of American and Chinese respondents. Figure 1 shows the statistical and conceptual framework of this study using all the above-mentioned measures. The LCA method was used to identify the latent parent–child relationship classes using seven observed indicators. LCA is a useful statistical technique for identifying latent population groupings with similar underlying characteristics based on a set of observed variables. By computing the item-response probabilities conditioned on latent class membership, LCA can group homogeneous respondents and identify the characteristics of each subgroup (Nylund-Gibson & Choi, 2018). In contrast to conventional unidimensional classification techniques, LCA is person centered and generates unique typologies illustrating the patterns of linkages between observed indicators (Nguena Nguefack et al., 2020). LCA has been widely used in studies examining the structure of social relationships (Guo et al., 2020; Silverstein & Bengtson, 1997).

The LCA model is estimated using the maximum-likelihood approach. Researchers can define the number of latent classes and choose the optimal model (Nylund-Gibson & Choi, 2018). The optimal number of latent classes is determined by multiple factors, including model-fitting indices and results interpretability (Lu et al., 2021; Nguena Nguefack et al., 2020). Model-fitting indices include the Bayesian information criterion (BIC), Akaike information criterion (AIC), size-adjusted Bayesian information criterion (aBIC), consistent Akaike information criterion (cAIC), likelihood-ratio test, and entropy (similar to pseudo *R*^2^; Niels, 2015). Among these, the BIC is recognized as the most reliable indicator (Weller et al., 2020). When determining the optimal number of classes, we also evaluated whether each latent class made sense in the research context. Once the optimal number of classes has been determined, each respondent is allocated to a new class based on posterior group probabilities.

Because older parents in the two nations are affected by distinct cultures, their latent parent–child relationship patterns could be different and each country may have unique classes specific to its own context (Guo et al., 2012; Silverstein & Bengtson, 1997). Therefore, we performed LCA separately for each country. As a sensitivity check, we pooled the two countries’ data together to re-estimate LCA.

Finally, we examined the associations between individuals’ new class memberships and their health statuses using regression models, controlling for the covariates (Figure 1). Linear regression models of depressive symptoms and functional limitations revealed violations of the residual normality assumption. These two variables were thus rescaled back to the original scale (Supplementary Table 1) and considered as count variables in the analysis. Due to the right-skewed distribution of these count variables, negative binomial regression was used to address over-dispersion. Linear regression was used to examine the association between class membership and cognitive function. The model assumptions were reasonably met in the cognitive function model. Data were assumed to be missing at random (Lu & Shelley, 2022); therefore, we used multiple imputation to address the missingness issue. The HRS and CHARLS sampling weights were not applied, because this study used a subset of the full sample and did not conduct population-level descriptive prevalence analysis (Solon et al., 2015). All analyses were conducted in R using the packages “polka” (Linzer & Lewis, 2011), “mice” (Zhang, 2016), and “MASS” (Venables & Ripley, 2002).

## Results

### Descriptive Characteristics

Significant between-country differences were found for the sample characteristics across all measures (*p*s < .001), except the number of grandchildren and financial transfer to children (Table 1). Chinese respondents were younger (mean age = 73, standard deviation [*SD*] = 6.43), had a higher proportion of males (53%), and were more likely to be married/partnered (59%) than their non-Hispanic White American counterparts (mean age = 78, *SD* = 7.94, 30% male, 37% married). The Chinese sample had much lower educational attainments than non-Hispanic White Americans: 92.43% of Chinese had less than upper secondary education, whereas only 14.42% of older non-Hispanic White Americans were in this category. Chinese respondents were also less likely to be retired (58%) than Americans (93% retired). Both Chinese and non-Hispanic White Americans had two people living in the household and five grandchildren on average. Many Chinese respondents had four children, whereas non-Hispanic White Americans had three on average. Chinese respondents also reported lower household income, poorer self-reported health, and fewer chronic diseases than their non-Hispanic White American counterparts.

**Table 1.** Descriptive Characteristic of Respondents in the United States (Non-Hispanic Whites Only) and China

Measures	United States (non-Hispanic Whites only)	Chinese	*P* value
Number of observations	3,918	4,058	
Demographic and health characteristics			
Age, mean ± *SD*	77.96 ± 7.94	72.88 ± 6.43	<.001
Sex, male, *n* (%)	1,185 (30.25)	2,137 (52.66)	<.001
Education, *n* (%)			<.001
Less than upper secondary	565 (14.42)	3,750 (92.43)	
Upper secondary and vocational training	2,484 (63.42)	227 (5.60)	
Tertiary	868 (22.16)	80 (1.97)	
Marital status, married/partnered, n (%)	1,434 (36.52)	2,400 (59.14)	<.001
Retired, yes, *n* (%)	3,499 (93.11)	2,281 (57.70)	<.001
Household size, median (IQR)	2 (1, 2)	2 (2, 3)	<.001
Number of children, median (IQR)	3 (2, 4)	4 (3, 5)	<.001
Number of grandchildren, median (IQR)	5 (3, 8)	5 (3, 8)	.07
Household income, mean ± *SD*	10.43 ± 0.99	6.67 ± 2.68	<.001
Self-reported health, mean ± *SD*	3.09 ± 1.06	2.93 ± 0.96	<.001
Number of chronic diseases, median (IQR)	3 (2, 4)	1 (1, 2)	<.001
Parent–child relationship indicators			
Coresidence, yes, *n* (%)	709 (18.13)	2,083 (51.33)	<.001
Live nearby, yes, *n* (%)	2,205 (57.68)	3,749 (93.58)	<.001
Have weekly contact, yes, *n* (%)	3,287 (83.89)	3,701 (91.41)	<.001
Receive instrumental assistance from children, yes, *n* (%)	459 (11.74)	871 (21.46)	<.001
Provide care to grandchildren, yes, *n* (%)	551 (14.06)	972 (23.95)	<.001
Financial transfer from children, yes, *n* (%)	268 (6.91)	3,496 (86.15)	<.001
Financial transfer to children, yes, *n* (%)	1,294 (33.47)	1,333 (32.85)	.57
Health statuses			
Depressive symptoms, mean ± *SD*	0.17 ± 0.24	0.43 ± 0.24	<.001
Functional limitations, mean ± *SD*	0.41 ± 0.28	0.34 ± 0.28	<.001
Cognitive function, mean ± *SD*	0.62 ± 0.15	0.43 ± 0.17	<.001

*Notes*: IQR = interquartile; *SD* = standard deviation. Reference level: female for sex, partnerless for marital status, no for retired, no for all parent–child relationship indicators. For continuous measures, mean ± *SD* was used to describe the distribution and *t*-test was used to compare the means between two countries. For categorical/binary measures, number (percent) was used to describe the distribution and chi-square test was used to compare the distribution between two countries. For count measures with highly skewed distribution, median (IQR) was used to describe the distribution and Wilcoxon rank-sum test was used to compare the medians between two countries.

Regarding parent–child relationships, the United States–China comparison revealed significant differences (*p*s < .001). Compared to older non-Hispanic White American parents, a greater proportion of older Chinese parents coresided with their children, lived near their children, had weekly contact, received instrumental assistance from their children, provided care to grandchildren, and received financial transfers from their children. A similar proportion of older non-Hispanic White Americans and Chinese provided financial support to their children (33%). For health statuses, Chinese respondents reported more depressive symptoms and poorer cognitive function than their U.S. non-Hispanic White counterparts but fewer functional limitations (mean = 0.34, *SD* = .28) than U.S. respondents (mean = 0.41, *SD* = .28).

### LCA Results

Table 2 presents the fitting indices of different latent class models in the two countries separately. In the U.S. non-Hispanic White sample, the four-class model achieved the lowest BIC (23,935.19), aBIC (23,836.69), and cAIC (23,966.19). The entropy of the four-class model (0.45) was very close to that of the three-class and five-class models. A qualitative review demonstrated that each of the four classes in the U.S. non-Hispanic White sample made sense in the study context. For the Chinese sample, the three-class model had the lowest BIC (25,572.71) and cAIC (25,595.71). The entropy in the three-class model (0.41) was the second largest. The qualitative evaluation revealed that the three-class model was suitable in the Chinese context. Therefore, the four-class model was chosen for non-Hispanic White American respondents, and the three-class model was selected for Chinese respondents.

**Table 2.** Fitting Indices of Latent Class Model Result in the United States (Non-Hispanic Whites Only) and China

Model	Log-likelihood	*df* of residuals	BIC	aBIC	cAIC	Likelihood ratio	Entropy
United States (non-Hispanic Whites only)							
1-Class	−12,112.9	120	24,283.56	24,261.32	24,290.56	686.71	—
2-Class	−11,976.4	112	24,076.43	24,028.76	24,091.43	413.67	0.40
3-Class	−11,901.3	104	23,992.16	23,919.08	24,015.16	263.49	0.47
**4-Class**	−**11,839.9**	**96**	**23,935.19**	**23,836.69**	**23,966.19**	**140.61**	**0.45**
5-Class	−11,823.5	88	23,968.35	23,844.43	24,007.35	107.85	0.46
China							
1-Class	−13,282.2	120	26,622.42	26,600.18	26,629.42	1,373.94	—
2-Class	−12,745.0	112	25,614.43	25,566.77	25,629.43	299.60	0.62
**3-Class**	−**12,691.0**	**104**	**25,572.71**	**25,499.63**	**25,595.71**	**191.51**	**0.41**
4-Class	−12,664.2	96	25,585.50	25,486.99	25,616.50	137.94	0.23

*Notes*: aBIC = size-adjusted Bayesian information criterion; AIC = consistent Akaike information criterion; BIC = Bayesian informal criterion; cAIC = Akaike’s Information Criterion; *df* = degree of freedom.

The row in bold was suggested to have the most optimal model fit after considering several indices.

**Figure 2.**
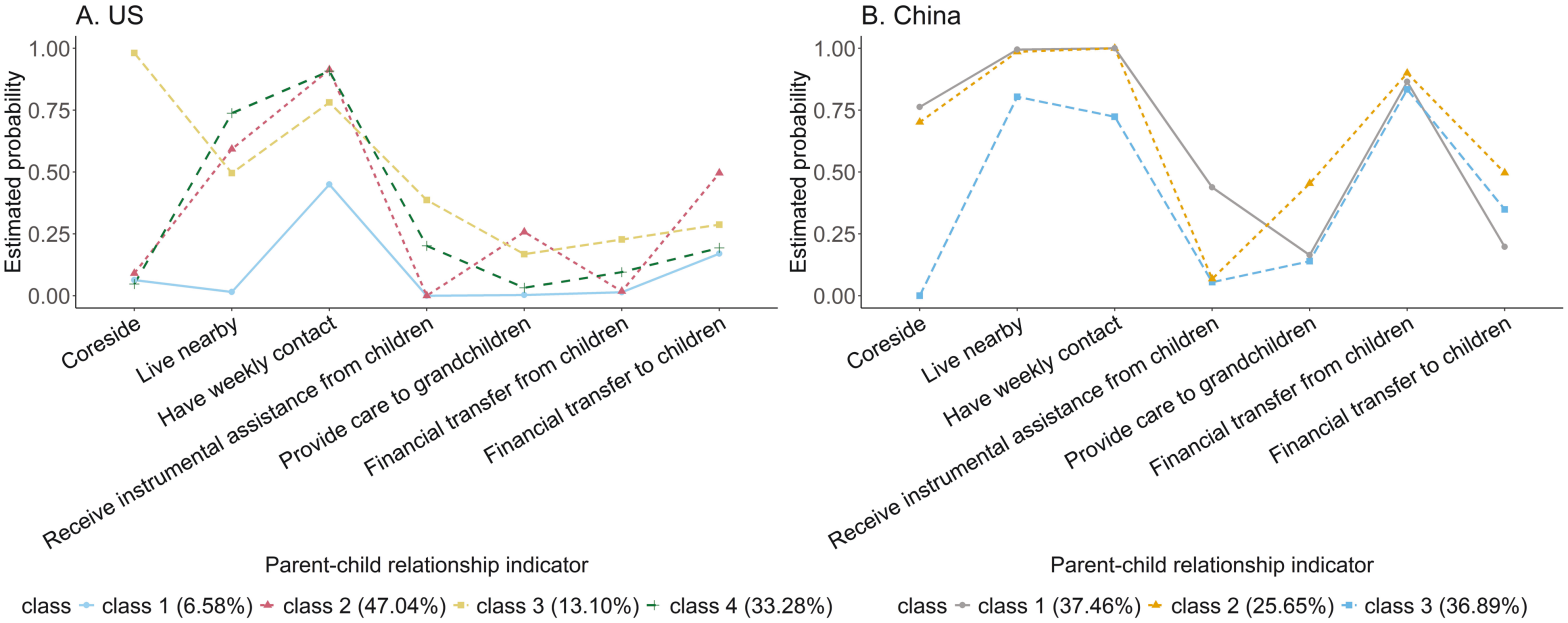
Statistical index plot for latent parent–child relationship classes in two countries.

For the non-Hispanic White U.S. sample, four distinct classes were identified (Table 3, Figure 2A), with the likelihood of having weekly contact being high in all classes.

**Table 3.** Conditional Item-Response Probabilities by Class

Indicator	Coreside	Live nearby	Have weekly contact	Receive instrumental assistance from children	Provide care to grandchildren	Financial transfer from children	Financial transfer to children	Defining characteristics
United States (non-Hispanic Whites only)								
Class 1 (6.58%)	0.06	0.02	**0.45**	0.00	0.00	0.01	0.17	Distant and uninvolved
Class 2 (47.04%)	0.09	**0.59**	**0.91**	0.00	**0.26**	0.02	**0.50**	Geographically proximate with frequent contacts and downward support
Class 3 (13.10%)	**0.98**	**0.50**	**0.78**	**0.39**	0.17	**0.23**	0.29	Coresident with frequent contacts and upward support
Class 4 (33.28%)	0.05	**0.74**	**0.91**	0.20	0.03	0.10	0.19	Geographically proximate with frequent contacts
China								
Class 1 (37.46%)	**0.76**	**0.99**	**0.99**	**0.44**	0.16	**0.86**	0.20	Coresident with frequent contacts and upward support
Class 2 (25.65%)	**0.70**	**0.99**	**0.99**	0.07	**0.45**	**0.90**	**0.50**	Coresident/interdependent
Class 3 (36.89%)	0.00	**0.80**	**0.72**	0.05	0.14	**0.83**	0.35	Geographically proximate with frequent contacts and upward financial support

*Notes*: The conditional item-response probabilities were for the “yes” category in each parent–child relationship indicator. The probabilities that were the highest or above 0.4 were in bold and used to define the characteristics of each class.

1. Distant and uninvolved (6.58%), defined by the absence of coresidence, geographic proximity, or any instrumental or financial transfers with their children.
2. Geographically proximate with frequent contact and downward support (47.04%), defined as individuals with a high probability of living nearby and having weekly contact. This group also had the highest probability of providing care for grandchildren (0.26) and financial support to children (0.50) across all U.S. classes.
3. Coresident with frequent contacts and upward support (13.10%), comprising individuals with a high probability of coresiding, living in proximity, and having weekly contact. This group was most likely to receive instrumental support (0.39) and financial transfers from their children (0.23).
4. Geographically proximate with frequent contact (33.28%), characterized by individuals living in proximity and having weekly contact with children.

For the Chinese sample, three classes were identified (Table 3, Figure 2B), all of which were characterized by a high probability of living nearby, having weekly contact, and receiving financial transfers from children.

1. Coresident with frequent contact and upward support (37.46%). This group had the highest probability of receiving instrumental assistance from children (0.44).
2. Coresident/interdependent (25.65%), characterized by those with the highest probability of providing grandchild care (0.45) and financial support to children (0.50).
3. Geographically proximate with frequent contact and upward financial support (36.89%).

Sensitivity analyses including all racial/ethnic groups in the U.S. sample revealed that the five-class model was optimal (Supplementary Table 2). However, the five latent classes were not distinct—there were overlaps between Classes 1 and 3, as well as between Classes 2 and 4 (Supplementary Table 3), making the results difficult to interpret in the study context. Another sensitivity analysis using pooled data showed that the five-class model was optimal, considering it had the second smallest BIC and high entropy (Supplementary Table 3). Further examination of the conditional item-response probabilities found slightly different latent class patterns compared to the separate analyses (Table 3). However, two latent classes in the pooled analysis had very small proportions (<5%) in two countries (Supplementary Table 4), which is not recommended in LCA practice (Nguena Nguefack et al., 2020). In sum, results from other models/methods differ from main analysis were not interpretable/usable given this study dataset. 

### Regression Model Results

Table 4 presents the association between new class memberships and the three health statuses. For non-Hispanic White American older adults, compared to the class that was *geographically proximate with frequent contact*, the class of *providing downward support* was associated with fewer functional limitations (incidence risk ratio [IRR] = 0.86, 95% confidence interval [CI] = 0.82, 0.90) and better cognitive function (β = 0.02, 95% CI = 0.01, 0.03). In contrast, for Chinese older adults, compared to the class that *was geographically proximate with frequent contact and upward financial support*, the class of *receiving instrumental support from children* was associated with more depressive symptoms (IRR = 1.11, 95% CI = 1.05, 1.17) and functional limitations (IRR = 1.28, 95% CI = 1.20, 1.37), and poorer cognitive function (β = −0.03, 95% CI = −0.04, −0.01). Another two marginally significant associations were also found—the U.S. non-Hispanic White distant and uninvolved class was associated with more functional limitations (IRR = 0.88) and the China coresident/interdependent class was associated with better cognition (β = 0.02) than their respective reference groups. In sensitivity analysis, we additionally controlled for rural/urban residence to account for differences in social welfare systems and resources in the Chinese models, and the key findings remained the same.

**Table 4.** Regression Model Results Examining the Association Between Latent Parent–Child Relationship Classes and Health Statuses

	Depressive symptoms		Functional limitation		Cognitive function	
Latent classes	Negative binomial regression		Negative binomial regression		Linear regression	
	IRR	95% CI	IRR	95% CI	β	95% CI
United States (non-Hispanic Whites only)						
Class: geographically proximate with frequent contacts	Ref		Ref		Ref	
Class: distant and uninvolved	1.00	(0.83, 1.19)	0.88^*^	(0.81, 0.96)	0.01	(−0.004, 0.03)
Class: geographically proximate with frequent contacts and downward support	0.99	(0.89, 1.09)	0.86^***^	(0.82, 0.90)	0.02^***^	(0.01, 0.03)
Class: coresident with frequent contacts and upward support	1.05	(0.89, 1.25)	0.97	(0.91, 1.04)	-0.01	(−0.03, 0.002)
China						
Class: geographically proximate with frequent contacts and upward financial support	Ref		Ref		Ref	
Class: coresident/interdependent	1.03	(0.98, 1.09)	0.94	(0.88, 1.00)	0.02^*^	(0.002, 0.03)
Class: coresident with frequent contacts and upward support	1.11^***^	(1.05, 1.17)	1.28^***^	(1.20, 1.37)	−0.03^**^	(−0.04, −0.01)

*Notes*: CI = confidence interval; IRR = incidence risk ratio. Models were adjusted for demographic (age, sex, education, marital status, retirement status, household size, number of children, number of grandchildren, household income) and health condition (self-reported health, number of chronic diseases).

^*^ *p* < .05. ^**^p < .01. ^***^p < .001.

## Discussion

To our knowledge, this is the first cross-cultural comparative study using two nationwide data sets in the United States and China to examine the latent typologies of multifaceted parent–child relationships and their associated health statuses. Cultural contrasts were evident in the typologies of parent–child relationships. For non-Hispanic White American older parents, their relationship with their adult children was primarily characterized by a high probability of having weekly contact across all classes. In comparison, all classes in the Chinese sample were characterized by a high probability of living nearby, having weekly contact, and receiving financial transfers from their children. Moreover, the various parent–child relationship typologies showed differing associations with health in the two countries.

Overall, parent–child relationships in China tended to be closer than those of U.S. non-Hispanic Whites. Most Chinese older adults either coreside with their children or live nearby. This aligns with prior findings that intergenerational resource transfers in China remain uninterrupted despite the expanding nuclear family structure (Croll, 2006). This finding also corroborates a prior study indicating that in contemporary China, intergenerational coresidence and living nearby are becoming more and more common as younger adult children seek assistance from their aging parents for childcare (Gan & Fong, 2020). In contrast, coresidence was not typical in the U.S. non-Hispanic White sample. Previous studies have indicated that coresidence in the United States tends to be driven by financial and physical considerations (e.g., economic crisis and poor health conditions; Keene & Batson, 2010).

Additionally, our findings illustrate cross-national variations in the flow of intergenerational assistance. That is, intergenerational resource transfer in China reflects mutual support (i.e., downward and upward support), both financially and instrumentally (Lin & Yi, 2013; Nauck & Suckow, 2006), whereas, in the United States, financial transfers are primarily from aging parents to adult children. This finding is in line with existing evidence that intergenerational relations in China have evolved from conventional filial piety (i.e., the perception of caring for aging parents as unidirectional transfers from adult children to aging parents) to reciprocal and symmetric exchanges between aging parents and adult children (Croll, 2006). Financial transfers from children may also serve as a safety net in times of financial difficulty for Chinese older adults (Zhu, 2016), and their involvement in caring for grandchildren may function as repayment for their children’s financial assistance (Chen & Silverstein, 2000).

In the U.S. non-Hispanic White sample, older parents provided financial support to their children more often than vice versa, a finding consistent with earlier U.S. studies (Berry, 2008). According to prior research, older Americans’ financial assistance to children, particularly those who are economically disadvantaged (e.g., lower income, single parent, younger children) and those in the transition to adulthood, has increased over the past several decades (Berry, 2008; Padgett & Remle, 2016).

Although our findings are not directly comparable to prior research due to the different study designs, several discrepancies with the existing evidence were observed. Unlike previous studies (e.g., Dykstra & Fokkema, 2011), we did not find a distant and supportive typology in the U.S. non-Hispanic White sample. However, it is important to point out that our results only reflect the relative prevalence of the main intergenerational relationship types, which is not exhaustive. It is possible that other forms of typologies are not as prevalent, and therefore not captured in LCA models. In the Chinese sample, unlike previous studies (Guo et al., 2012, 2018), our results did not identify a distant relationship typology, despite the documented increase in China’s internal migration (Cong & Silverstein, 2011). It is possible that although the Chinese population has experienced a high migration rate, adult children coresiding and living nearby remain common. Our results suggest that over 51% of Chinese older adults coreside with their children. Nearly 37% of the sample were classified as geographically proximate with frequent contact and upward financial support (Class 3). This finding corroborates a prior study indicating that living in the same city but in different households has become more prevalent in contemporary China (Gan & Fong, 2020). We postulate that Class 3 represents an adapted filial piety in China (i.e., non-coresiding yet geographically proximate with frequent contact and upward financial support), which is comparable to the *modified extended family* prototype proposed in earlier research (Litwak, 1960).

Importantly, the typologies of parent–child relationships have different health implications in the two countries. In the U.S. non-Hispanic White sample, compared to the class that was geographically proximate with frequent contact, the class of providing downward support (i.e., caring for grandchildren and financial transfer to children) was associated with better health statuses (i.e., fewer functional limitations and better cognitive function). This finding could be partially explained by role enhancement theory. According to this theory, as older adults gain satisfaction and pleasure from social roles, their engagement in multiple social roles has health-promoting effects (Moen et al., 1995). As grandparents, older adults caring for grandchildren may strengthen their family ties, keep them cognitively stimulated and physically active, and give them a sense of purpose and competence, all of which can help improve their health (Di Gessa et al., 2016; Winefield & Air, 2010). However, due to the cross-sectional design of the study, we could not rule out the possibility of reverse causation. That is, healthier older adults are more likely to be involved in providing downward support than their less healthy peers.

In contrast, for Chinese older adults, compared to the class that was geographically proximate with frequent contact and upward financial support, the class of coresiding and receiving instrumental support from children was associated with poorer health conditions. We postulate that culturally, it is possible that older Chinese adults who receive asymmetrical instrumental support from children may be concerned about becoming a burden to their children, leading to decreased self-efficacy and psychological damages, which in turn link to poorer health conditions (Chen & Silverstein, 2000; Park et al., 2005). Another potential explanation is that the increased grandparent caregiving demands and intergenerational conflicts experienced by Chinese older adults who reside with their children negatively affect their health (Lu et al., 2021). However, it is also possible that Chinese older adults with poorer health are more likely to coreside with and obtain care from their adult children.

The associations between intergenerational relationships and health in the two countries indicate that autonomy may have an influence in both cultures. In the United States, providing downward support is linked to better health outcomes, whereas in China, intergenerational coresidence and receiving upward support are negatively associated with health. We postulate that non-Hispanic White older Americans who can offer assistance to younger generations are more likely to be self-reliant and independent, which in turn is associated with better health. In contrast, older Chinese individuals receiving support from their children may indicate dependence and a lack of autonomy, which could be associated with poorer health outcomes. The shared influence of autonomy in these two cultures aligns with its acknowledgment as an essential element of successful aging (Ford et al., 2000; Tesch-Römer & Wahl, 2016).

### Limitations

Several limitations of this study warrant discussion. First, although the HRS and CHARLS were based on nationwide samples, we applied some restrictions to the sample and did not use sampling weights. Furthermore, because our study focused exclusively on non-Hispanic White older adults, the results may not apply to other racial or ethnic groups due to the diversity of cultures in the U.S. population. Future research should include a more racial/ethnic diverse sample, including Americans with East Asian and Latino origins, to explore their parent–child relationships (Campos & Kim, 2017).

Second, the parent–child relationships in this study did not measure all key dimensions specified by the intergenerational solidarity model, such as affectual, consensual, and normative dimensions, and intergenerational ambivalence due to the unavailability of such measures in both datasets (Guo et al., 2020; Silverstein & Bengtson, 1997; Silverstein & Giarrusso, 2010). Because we used different indicators of intergenerational relationships from previous studies, our results were not directly comparable to those of other studies.

Third, the cross-sectional design limited our ability to determine causality, as well as to understand potential changes in parent–child relationships over time. It is possible that the poor health of older parents may lead to changes in intergenerational relationships (e.g., moving nearby and receiving instrumental support from children). Because the cross-sectional data were collected in 2014/2015, our study findings were also specific to the societal context of this particular period. Updated and/or longitudinal data are needed to extend our understanding of how the two countries’ parent–child relationship patterns evolve or change over time. Fourth, the LCA results are exploratory in nature and require further study to cross-validate our findings.

### Implications

The differences in parent–child relationships call for culturally relevant strategies for health promotion. For instance, our results indicate that coresidence and living nearby are dominant intergenerational living arrangements in China, but Chinese older adults who coreside with their children and receive financial and instrumental support from them may experience negative health outcomes. Therefore, family-based care programs (e.g., family caregiver support, home-based care services, and respite care) that maintain culturally informed care preferences while reducing their sense of burdening their family members are recommended. For non-Hispanic White American older adults, considering the positive health implications of grandparenting, programs related to grandparenting education and skill training may help them enjoy the grandparenting experience and ultimately promote their health. Additionally, future research using longitudinal modeling techniques such as latent transition analysis to examine dynamic transitions of parent–child relationships is recommended. Future longitudinal studies could also investigate the possible reverse pathway from health to intergenerational relationships.

## Conclusion

Cross-cultural differences in parent–child relationships and associated health statuses are evident. Overall, parent–child relationships in China tend to be closer than those of U.S. non-Hispanic Whites. Coresidence and receiving financial support from children are distinctive features of parent–child relationships in China. Caring for grandchildren and financial transfer to children were linked to positive health conditions among non-Hispanic White older Americans. Our findings should be tested in different cultural contexts, and they call for culturally relevant strategies to improve parent–child relationships and ultimately promote health among older adults.

## Supplementary Material

igae050_suppl_Supplementary_Materials

## Data Availability

This study used secondary data and was not preregistered. The HRS and CHARLS data are available to the public. For more information, please refer to the HRS and CHARLS webpages.
