# Supplementary material for: Parent–Child Relationship Typologies and Associated Health Status Among Older Adults in the United States and China: A Cross-Cultural Comparison
Source: Innov Aging. 2024 May 18;8(6):igae050. doi: 10.1093/geroni/igae050 (PMC11192862; doi:10.1093/geroni/igae050)
Supplement: igae050_suppl_Supplementary_Materials [file igae050_suppl_supplementary_materials.docx]

***Innovation in Aging* Supplementary Material: Kong et al. Parent–child relationship typologies and associated health status among older adults in the United States and China: A cross-cultural comparison.**

**Supplemental Table 1** Measures used in two countries and their coding and rescaling methods

| **Measures** | **US** | **China** | **Coding** | **Rescaling method** |
| --- | --- | --- | --- | --- |
|  | Definition/number of items | Definition/number of items |  |  |
| ***Parent-child relationship indicators*** | |  |  |  |
| Co-resided with their children |  |  | 1=yes, 0=no |  |
| Lived nearby their children | within 10 miles | the same city/county | 1=yes, 1=no |  |
| Had weekly contact with their children, in Person, by Phone, Mail or E-mail |  |  | 1=yes, 2=no |  |
| Received assistance from their children |  |  | 1=yes, 3=no |  |
| Provided grandchild care to their children |  |  | 1=yes, 4=no |  |
| Received any financial transfer from their children |  |  | 1=yes, 5=no |  |
| Provided any financial transfer to their children |  |  | 1=yes, 6=no |  |
| ***Health outcomes*** |  |  |  |  |
| Depressive symptom | 8-item | 10-item | US: range 0-8  China: range 0-30 | US: divided by 8  China: divided by 30  New range 0-1 |
| Functional limitations | 12-item | 9-item | US: range 0-12  China: range 0-9 | US: divided by 12  China: divided by 9  New range 0-1 |
| Cognitive function | 20-item memory + 15-item mental status | 20-item memory + 10-item mental status | US: range 0-35  China: range 0-30 | US: divided by 35  China: divided by 30  New range 0-1 |
| ***Covariates*** |  |  |  |  |
| Age |  |  | In years, continuous |  |
| Sex |  |  | Female vs. male |  |
| Education |  |  | Less than upper secondary vs. upper secondary and vocational training vs. tertiary |  |
| Marital status |  |  | Married/partnered vs. partnerless |  |
| Retirement status |  |  | Yes vs. no |  |
| Self-reported health |  |  | Ordinal, from 1=very poor to 5=very good |  |
| Number of chronic diseases |  |  | Range 0-8 |  |
| Household size |  |  | Count |  |
| Number of children |  |  | Count |  |
| Number of grandchildren |  |  | Count |  |
| Household income |  |  | Continuous | first transformed into purchasing power in USD, then log-transformed |

**Supplemental Table 2** Fitting indices of latent class model result among US respondents (Non-Hispanic White vs. all racial/ethnic groups)

| Model | log-likelihood | Df of residuals | BIC | aBIC | cAIC | likelihood-ratio | Entropy |
| --- | --- | --- | --- | --- | --- | --- | --- |
| ***US (Non-Hispanic White sample only)*** | | | | | | | |
| 1-Class | -12112.9 | 120 | 24283.56 | 24261.32 | 24290.56 | 686.71 | - |
| 2-Class | -11976.4 | 112 | 24076.43 | 24028.76 | 24091.43 | 413.67 | 0.40 |
| 3-Class | -11901.3 | 104 | 23992.16 | 23919.08 | 24015.16 | 263.49 | 0.47 |
| **4-Class** | **-11839.9** | **96** | **23935.19** | **23836.69** | **23966.19** | **140.61** | **0.45** |
| 5-Class | -11823.5 | 88 | 23968.35 | 23844.43 | 24007.35 | 107.85 | 0.46 |
| ***US (all racial/ethnic samples)*** | | | | | | | |
| 1-Class | -17843.1 | 120 | 35746.35 | 35724.11 | 35753.35 | 1035.21 | - |
| 2-Class | -17627.9 | 112 | 35384.43 | 35336.77 | 35399.43 | 604.64 | 0.48 |
| 3-Class | -17528.7 | 104 | 35254.79 | 35181.71 | 35277.79 | 406.35 | 0.44 |
| 4-Class | -17428.6 | 96 | 35123.21 | 35024.7 | 35154.21 | 206.12 | 0.44 |
| **5-Class** | **-17389.5** | **88** | **35113.72** | **34989.79** | **35152.72** | **127.98** | **0.42** |
| 6-Class | -17372.9 | 80 | 35149.13 | 34999.78 | 35196.13 | 94.74 | 0.40 |

Notes. The row in bold was suggested to have the most optimal model fit after considering several indices.

**Supplemental Table 3** Conditional item response probabilities by class among US respondents (Non-Hispanic White vs. all racial/ethnic groups)

| Indicator | Coreside | Live nearby | Have weekly contact | Receive instrumental assistance from children | Provide care to grandchildren | Financial transfer from children | Financial transfer to children | Defining characteristic |  |
| --- | --- | --- | --- | --- | --- | --- | --- | --- | --- |
| ***US (Non-Hispanic White sample only)*** | | | | | | | | | |
| Class 1 (6.58%) | 0.06 | 0.02 | **0.45** | 0.00 | 0.00 | 0.01 | 0.17 | distant and uninvolved |  |
| Class 2 (47.04%) | 0.09 | **0.59** | **0.91** | 0.00 | **0.26** | 0.02 | **0.50** | geographically proximate with frequent contacts and downward support |  |
| Class 3 (13.1%) | **0.98** | **0.50** | **0.78** | **0.39** | 0.17 | **0.23** | 0.29 | co-resident with frequent contacts and upward support |  |
| Class 4 (33.28%) | 0.05 | 0.74 | **0.91** | 0.20 | 0.03 | 0.10 | 0.19 | geographically proximate with frequent contacts |  |
| ***US (all racial/ethnic samples)*** | | | | | | | | | |
| Class 1 (22.7%) | 0.12 | 0.14 | **0.62** | 0.01 | 0.00 | 0.03 | 0.20 | distant and uninvolved |  |
| Class 2 (6.96%) | **0.63** | **0.54** | **0.84** | 0.06 | **0.53** | **0.26** | **0.46** | geographically proximate with frequent contacts and downward support |  |
| Class 3 (40.62%) | 0.18 | **0.78** | **0.93** | 0.23 | 0.04 | 0.11 | 0.17 | geographically proximate and uninvolved |  |
| Class 4 (25.89%) | 0.03 | **0.64** | **0.92** | 0.00 | **0.27** | 0.00 | **0.50** | geographically proximate with frequent contacts and downward support |  |
| Class 5 (3.83%) | **0.99** | **0.41** | **0.78** | **0.77** | 0.05 | **0.24** | 0.16 | co-resident with frequent contacts and upward support |  |

Notes. The row in bold was suggested to have the most optimal model fit after considering several indices.

**Supplemental Table 4** Fitting indices of latent class model result among pooled respondents (US Non-Hispanic White+China)

| Model | log-likelihood | Df of residuals | BIC | aBIC | cAIC | likelihood-ratio | Entropy |
| --- | --- | --- | --- | --- | --- | --- | --- |
| 1-Class | -29752.98 | 120.00 | 59568.68 | 59546.44 | 59575.68 | 3843.90 | - |
| 2-Class | -28274.07 | 112.00 | 56682.56 | 56634.89 | 56697.56 | 886.09 | 0.619 |
| 3-Class | -28115.65 | 104.00 | 56437.40 | 56364.31 | 56460.40 | 569.24 | 0.81 |
| 4-Class | -28073.70 | 96.00 | 56425.18 | 56326.67 | 56456.18 | 485.34 | 0.686 |
| **5-Class** | **-27983.62** | **88.00** | **56316.70** | **56192.77** | **56355.70** | **305.18** | **0.718** |
| 6-Class | -27946.62 | 80.00 | 56314.39 | 56165.04 | 56361.39 | 231.19 | 0.589 |
| 7-class | -27921.25 | 72.00 | 56335.33 | 56160.56 | 56390.33 | 180.44 | 0.625 |

Notes. The row in bold was suggested to have the most optimal model fit after considering several indices.

**Supplemental Table 5** Conditional item response probabilities by class among pooled respondents (US Non-Hispanic White + China)

| Classes | Distribution  (Overall) | Coreside | Live nearby | Have weekly contact | Receive instrumental assistance from children | Provide care to grandchildren | Financial transfer from children | Financial transfer to children | Defining characteristic | Distribution  (US) | Distribution  (China) |
| --- | --- | --- | --- | --- | --- | --- | --- | --- | --- | --- | --- |
| Class 1 | 5.13% | 0.13 | 0.16 | 0.00 | 0.00 | 0.07 | 0.19 | 0.21 | distant and uninvolved | 8.19% | 2.25% |
| Class 2 | 5.97% | 0.40 | **0.72** | **0.86** | **1.00** | 0.02 | 0.24 | 0.19 | geographically proximate with frequent contacts and upward support | 10.04% | 2.12% |
| Class 3 | 17.59% | 0.00 | **0.95** | **0.82** | 0.09 | 0.17 | **0.92** | **0.37** | geographically proximate with frequent contacts and primarily financial interaction | 2.14% | 32.19% |
| Class 4 | 43.54% | 0.13 | **0.62** | **0.93** | 0.01 | 0.16 | 0.12 | **0.36** | geographically proximate with frequent contacts and downward support | 76.28% | 12.59% |
| Class 5 | 27.77% | **0.86** | **0.99** | **1.00** | 0.29 | **0.30** | **0.90** | 0.32 | Co-resident and two-way support | 3.35% | 50.85% |

Notes. The row in bold was suggested to have the most optimal model fit after considering several indices.
